# Minimizing Risk of Nephrogenic systemic fibrosis in Cardiovascular Magnetic Resonance

**DOI:** 10.1186/1532-429X-14-31

**Published:** 2012-05-20

**Authors:** Theresa Reiter, Oliver Ritter, Martin R Prince, Peter Nordbeck, Christoph Wanner, Eike Nagel, Wolfgang Rudolf Bauer

**Affiliations:** 1 Department of Internal Medicine I, Divisions of Cardiology and Nephrology, University Hospital Wuerzburg, Wuerzburg, Germany; 2 Department of Radiology, Cornell & Columbia Universities, New York, USA; 3 Division of Imaging Sciences, King’s College London, London, UK

## Abstract

Nephrogenic Systemic Fibrosis is a rare condition appearing only in patients with severe renal impairment or failure and presents with dermal lesions and involvement of internal organs. Although many cases are mild, an estimated 5 % have a progressive debilitating course. To date, there is no known effective treatment thus stressing the necessity of ample prevention measures. An association with the use of Gadolinium based contrast agents (GBCA) makes Nephrogenic Systemic Fibrosis a potential side effect of contrast enhanced magnetic resonance imaging and offers the opportunity for prevention by limiting use of gadolinium based contrast agents in renal failure patients. In itself toxic, Gadolinium is embedded into chelates that allow its safe use as a contrast agent. One NSF theory is that Gadolinium chelates distribute into the extracellular fluid compartment and set Gadolinium ions free, depending on multiple factors among which the duration of chelates exposure is directly related to the renal function. Major medical societies both in Europe and in North America have developed guidelines for the usage of GBCA. Since the establishment of these guidelines and the increased general awareness of this condition, the occurrence of NSF has been nearly eliminated. Giving an overview over the current knowledge of NSF pathobiochemistry, pathogenesis and treatment options this review focuses on the guidelines of the European Medicines Agency, the European Society of Urogenital Radiology, the FDA and the American College of Radiology from 2008 up to 2011 and the transfer of this knowledge into every day practice.

## Review

Cardiovascular Magnetic Resonance (CMR) has gained an increasingly important role among the diagnostic methods due to its superb soft tissue imaging qualities without ionizing radiation and minimal risks. CMR is one of the uprising areas of MRI offering deep insight in both cardiac structure and function which minimizes the risks of failing to make an accurate diagnosis or resorting to more invasive tests [1]. Use of contrast enhanced imaging techniques allows the assessment of perfusion, tissue viability and detailed angiographic studies. The overwhelming majority of these techniques use Gadolinium based contrast agents (GBCAs). From the early days of GBCA enhanced imaging, these contrast agents were considered safe with only rare allergic reactions or local irritation from extravasations. However, in 1997, a new disease emerged, originally called Nephrogenic Fibrosing Dermopathy. It was first described by Cowper in 2001 [2] and the relationship to GBCA exposure has been strongly suspected since 2006 [3-7]. Later this entity was renamed Nephrogenic Systemic Fibrosis (NSF) when involvement of internal organs was discovered. An NSF Registry, www.icnfdr.org documented over 355 cases that all occurred in patients on dialysis or with severe renal dysfunction [8]. However since the connection between NSF and GBCAs has become known changes in MRI protocols with the focus on prevention has led to a decrease in NSF incidence. Reports are showing virtually no new NSF cases since 2008 in both patients with normal renal function and patients with renal impairment [9-11] in spite of continued use of GBCA, albeit at lower doses.

Here we review the clinical features of NSF and show how to use GBCA safely in patients at risk for NSF.

### Pathobiochemistry of gadolinium

Gadolinium is one of the 14 elements of the lanthanide group. Virtually all of its compounds contain it as the paramagnetic Gd^3+^ ion which has seven unpaired electrons in its half-filled 4 f outer shell. Gd^3+^ has a long electronic relaxation time based on its totally symmetric S state making it well suited for use as an MR contrast agent. It accelerates the relaxation of the water molecules present in the tissue, giving rise to an enhanced signal on T1-weighted images and, together with appropriate sequence parameters, an improved image contrast. However, gadolinium, like most metals, interferes with the complex biochemical processes of living organisms. In particular, it can act as a competitive inhibitor of calcium ions due to its large ionic radius (0.97 Å vs. 1.06 Å for Ca^2+^) and its high ionic charge. As a result various physiological processes involving Ca^2+^ can be influenced by the presence of Gd^3+^ such as Ca^2+^-activated ATPase in the sarcoplasmatic reticulum of skeletal muscle fibres, the reticuloendothelial system and some other enzymes such as dehydrogenases and glutathione S transferases. It is also known that Gd^3+^ has an inhibitory effect on Kupffer cells [4].

The toxic effects of Gd^3+^ can be suppressed by encasing it in an organic chelator. A variety of such chelators have been FDA/EMEA approved for use in patients and others are still being investigated [12-14]. The contrast agents in clinical use are based on the linear ionic chelator diethylenetriamine pentaacetic acid (often dubbed as DTPA or "pentetic acid"), the linear non-ionic chelator benzyloxyproprionictetra- acetate (“BOPTA”) or on the cyclic ionic chelator tetraazacyclododecane tetraacetic acid (DOTA). Other cyclic non-ionic chelators are tetraazacycl ododecane (DO3A). Stability of these agents is characterized in two ways: Thermodynamic stability describes the tendency of the chelate to dissociate into its components given an unlimited amount of time. It is expressed numerically as the logarithm of the stability constant. Kinetic stability describes the timescale of the dissociation expressed either as a rate constant or a half-life. Both characteristics depend on the surrounding milieu: Decreasing pH and increasing temperature favour dissociation [15]. As a rule, the macrocyclic chelates are several orders of magnitude more stable with regard to dissociation and transmetalation than their linear counterparts [16-18]. Furthermore, the presence of ions such as Ca^2+^, Cu^2+^ or Zn^2+^ which can replace Gd^3+^ from the chelate in a transmetalation reaction promote the unwanted release of Gd^3+^ exposing tissues to its toxic effects [19]. Indeed, early in vivo studies using radioisotope labelled Gd chelates indicate a relationship between kinetic stability and tissue-uptake of gadolinium [20].

**Figure 1:**
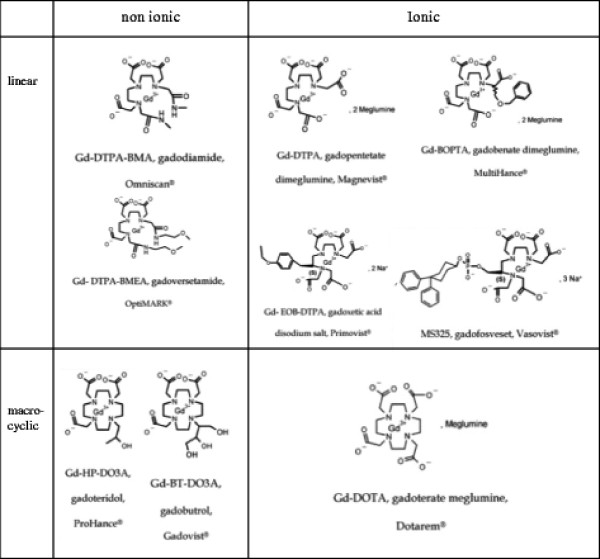
**Molecular structures of all currently available Gadolinium- based contrast agents.** The three agents in the lower section (asterisk) are presently considered the agents with highest safety due to their macrocyclic structure.

Besides the characterization in terms of macrocyclic and linear chelate structures, the nine currently available GBCAs (Table 1, Figure 1) can also be categorized by ionicity. Non-ionic Gd^3+^ chelates cause less osmotic stress because they do not require counterions such as Na^+^ in their formulations. They are closer to ionic neutrality with lower viscosity, and are less hydrophilic than ionic Gd^3+^ chelates [21]. As with iodinated contrast, non-ionic Gd^3+^ chelates appear to have a lower rate of allergic reactions [22]. Unfortunately non-ionic linear chelates are also less stable than their negatively charged analogs. With the exception of three linear ionic GBCAs that have lipophilic groups in their chelate structures (Gadobenate, Gadoxetate, Gadofosvescet), GBCAs are eliminated exclusively via the renal pathway. The estimated half-life of renal elimination of GBCAs for patients with normal renal function is about 90 min. However, with decreasing renal function the effective half-life increases to up to 18 – 34 hours [16,23]. Within this timescale gadolinium release for the linear chelates may be significant [20].

**Table 1:** Stability of Gadolinium- based magnetic resonance imaging contrast agents in human serum at 37°C, K(therm)

**Class**	**Net Charge**	**Trade Name**	**Short Names**	**Log K(therm)**	**Log K(cond7.4)**	**Kinetic Stabiltiy**
Linear	Non-ionic	Omniscan	Gadodiamide	16.9	14.9	Low
	Non-ionic	Optimark	Gadoversetamide	16.8	15.0	Low
	Ionic	Magnevist	Gadopentetate	22.5	18.4	Medium
	Ionic	Multihance	Gadobenate	22.6	18.4	Medium
	Ionic	Primovist	Gadoxetate	23.5	18.7	Medium
	Ionic	Vasovist	Gadofosvescet	22.1	18.9	Medium
Macrocyclic	Non-ionic	Gadovist	Gadobutrol	21.8	15.5	High
	Non-ionic	Prohance	Gadoteridol	23.8	17.2	High
	Ionic	Dotarem	Gadoterate	25.6	19.3	High

Stability constants measured at high basic pH. K(cond7.4) describe the complex stability constant at physiological conditions (pH 7.4). The values of K(cond7.4) are smaller than the values of K(therm). Smaller values indicate less complex stability [24][21]. The Values of K(therm) and K(cond7.4) are based on studies of Kumar, Cacheris, Toth, Imura, Schmitt-Willich, Uggeri, Caravan and Moreau [12,25-31]. Kinetic stability describes the activation energy required to break the chelate-Gadolinium bond.

Two of the three mentioned linear, ionic GBCAs (Gadobenate, Gadoxetate) have an aromatic component within the chelate structure that allows hepatocellular uptake and partial excretion via the biliary pathway. The third mentioned GBCA (Gadofosveset) has a biphenylcyclohexyl group that reversibly binds to albumin, extending the plasma half-life to about 18.5 hours for patients with a normal renal function [16,19].

### Pathogenesis of nephrogenic systemic fibrosis

NSF occurs in patients with acute or chronic renal failure [32,33]. The vast majority of NSF cases (approximately 95 %) have occurred in renal failure patients who received GBCA enhanced CMR imaging techniques prior to symptom onset [34]. Thus, it is likely that GBCAs play a role in triggering NSF. All GBCA stimulate the proliferation of fibroblasts, the linear GBCAs show a more potent stimulation than macrocyclic GBCAs [35-38] which raises the possibility that GBCA dissociation from the chelator is not necessary for NSF to occur. One hypothesis is that macrophages phagocytose the Gd^3+^ complexes that then, being located in intracellular lysosomes, stimulate the production of cytokines, and growth factors [39,40]. Local inflammation may be due to local Gd^3+^ deposition, that is triggered by local CD68+ or XIIIa+ dendritic cells, and a systemic inflammatory response that is associated with CD34+ fibrocytes that originate from the bone marrow [41]. The heterogeneous phenotypes of cells found in NSF lesion imply that both local and systemic inflammatory mechanisms might coexist [42]. TGF-beta1 levels are elevated in patients with NSF, and some studies have shown increased decortin levels, alpha-smooth muscle actin and hyaluron synthesis [38,43-45]. A modulation of collagen syntheses with an increase of Collagen I and III production as well as an increase in fibronectin expression has been documented [38,46], as well as increased VEGF levels, Ostepontin, and TIMP-1 expression [47]. At least for one of the GBCAs (Gadodiamide), an effect on the expression of chemokine genes has been tracked down as the presence of GBCAs increase the activation of a NFκB pathway. It shows that exposure to GBCAs and the included Gd^3+^ is a potent stimulator of normal macrophages [48]. Most recent studies suggest that possibly other cells might be involved in the development of NSF as well, such as tissue monocytes and macrophages in the peripheral blood [49].

Additionally, it has been discussed that pro-inflammatory events, like vascular thrombosis, myocardial infarction, trauma, sepsis or recent surgery might contribute to the development of NSF [33]. Sepsis might even trigger the onset of NSF without exposure to GBCAs [50]. A resent work showed that the presence of GBCA increases DNA damage in lymphocytes [51]. The presence of high iron and erythropoetin levels are suspected to contribute to the development of NSF as well [44,52-54]. The Gd^3+^ complexes seem to have an effect on calcium phosphate as a study showed that the calcium phosphate precipitation is increased thus activating macrophages [55].

### Symptoms, diagnosis and differential diagnosis

Patients with NSF present with skin lesions typically beginning on the distal extremities starting with indurated plaques and papules especially on edematous lower extremities, later on the upper extremities, trunk and eye section develop over days to several weeks and later show a woody texture. The plaques are described as brawny, and the skin may develop hyperpigmentation [56][57]. NSF lesions usually occur symmetrically. Patients may report sharp pain as well as pruritus, causalgias and paresthesias in afflicted areas. Stiffness and joint contractures can lead to decreased mobility. The progression is rapid in an estimated 5 % of cases [8] leading to immobility within weeks and in a few instances death has been attributed to NSF. Besides skin developing lesions, internal organs can be afflicted including lung, heart, liver, bones and kidneys [33,34].

NSF diagnosis is usually postulated upon the medical history/physical exam and confirmed with a deep punch skin biopsy [2,56,58,59]. Eosin and hematoxylin staining are used for demonstrating the typical features. The skin biopsy shows a dermal fibrosis with a high density of CD 34 positive and procollagen I positive fibrocytes (circulating fibrocytes) and collagen bundles with prominent clefts between the bundles. Besides these collagen bundles elastic fibres can be detected as well. Additionally, but not required for diagnosis of NSF, factor XIIIa positive dendritic cells may be detected [56]. However the histopathological and clinical features can overlap with other entities. Among these are Lipodermatosclerosis, Scleroderma and Morphea, Scleromyxedema, Porphyria cutanea tarda, Spanish toxic Oil syndrome, eosinophilic fasciitis and Eosinophilia- myalgia syndrome and chronic graft versus host disease [2,58,60]. It is necessary to combine both the clinical features and the histopathologic findings in order to avoid misdiagnosis.

Exposure to gadolinium might arouse suspicion towards the diagnosis of NSF however it has to be stressed that exposure to GBCAs does not factor into the diagnosis. The lack of GBCA exposures does not exclude NSF as diagnosis, as in about 5 % of NSF patients no GBCA exposure prior to symptom onset could be found.

Besides the development of NSF other more acute reactions to GBCA exposure have been described. Symptoms that imply the development of septicaemia have been described within 12-36 h after administration of GBCA [61]. Allergic reactions albeit rare are another GBCA risk [22][62]. The possibility of deterioration in renal function after GBCA exposure in renal failure patients is controversial but in the usually administered dosages GBCAs are less nephrotoxic than iodinated contrast agents even with the high doses used for MR viability imaging and MR Angiography [63,64].

### Incidence and prevalence

The incidence of NSF prior to 2008 varied widely among different institutions ranging from 0.26 % in patients on dialysis without any contributing factors to up to 8.8 % in patients with a eGFR smaller than 15 ml/min/1.73 m³ without hemodialysis [50,65]. A study published in 2007 in another center calculated an absolute risk for developing NSF in patients on chronic dialysis of 2.4 % per GBCA enhanced study and an absolute risk of 3.4 % per patient [66]. The combination of renal insufficiency and proinflammatory processes (e.g. operations, thrombembolic events, endothelial/vascular injury) adds up to an NSF incidence of 4.6 % [33]. The incidence is further increased when renal failure patients on dialysis develop sepsis, and has been estimated at 6.3 % [50]. It is also noteworthy that the overwhelming majority of cases was either on dialysis or had an eGFR < 15 ml/min/1,73 m³. Only a handful of cases estimated in patients with an estimated GFR > 30 ml/min/1,73 m³and most of these were patients in acute renal failure where GFR estimation is unreliable. The incidence of reported NSF cases in patients with a normal renal function is zero.

The vast majority of NSF cases have been reported in patients who underwent GBCA enhanced imaging however, about 5 % of NSF cases showed no traceable exposure to GBCAs prior to NSF onset [34]. The exposure to GBCAs seems to be a cofactor to developing NSF, with the incidence depending besides the already mentioned patients collective also on the type and dose of GBCA. In patients who received a GBCA dose according to the labeling (e.g. 0.1 mmol/kg) overall incidence is near zero, regardless of their renal function. Based on a report of Prince (2008, [65]) the use of higher GBCA doses such as double or triple dose GBCA administration (0,2 – 0,3 mmol/kg) increases the incidence from zero to 0,17 % for some of the available contrast agents. These were Gadodiamide and Gadobenate, while this effect was not detected with Gadopentetate dimeglumine and Gadoteridol. The incidence rises when GBCAs in high doses are used in patients with renal failure. One of the reports describes the incidence in this setting at 8.0 % [65,67-69].

The majority of reported cases are associated with non-ionic linear contrast agents (Gadodiamide or Gadoversetamide). Another agent associated with NSF is Gadopentate dimeglumine, but there are markedly fewer cases with Gadopentate dimeglumine than with Gadodiamide in spite of Gadopentetate dimeglumine having greater market share. Based on data presented at the FDA Joint Meeting of the Cardiovascular and Renal Drugs and Drug Safety Advisory Committee (Dec. 2009, observed time frame 2005–2009), 382 cases of NSF are related to the administration of Gadodiamide (estimated doses 13 million worldwide), 195 cases related to the administration of Gadopentetate dimeglumine (estimated doses 23 million doses worldwide) and 35 cases are attributed to Gadoversetamide (estimated 4.7 million doses worldwide). To date, no cases have been associated with the administration of Gadoxetate or Gadofosveset [70].

The American NSF registry has documented over 355 proven cases of NSF so far, however, other groups have reported deviant numbers, e.g. Zou et al. report of 408 cases that were biopsy confirmed [71]. Most likely, not all cases have been reported to the NSF registry, but directly to the FDA or not at all.

NSF is rarely seen in pediatric patients [72-74]. The youngest known case of NSF is a 6 year old patient even though many newborn babies with immature kidneys in the past received high doses of GBCA for multiple MR scans to assess congenital heart disease. This suggests that infants and newborns may be a protected population [63].

### Treatment of nephrogenic systemic fibrosis

Many NSF patients have improved or even been cured with restoration of normal renal function. This has occurred when acute renal failure resolves and with renal transplantation. Otherwise, there is no proven effective therapy for the treatment of NSF and to date the treatment options are limited to symptomatic relief. Physical therapy supposedly improves the range of motion [75]. Pain medication usually is needed and includes the use of opioids, NSAR´s, steroids and antidepressants. A single case reports that acetazolamide showed a good pain relieving effect in a meningeal affection of NSF. In some cases intravenous procainamid has been shown to suppress the nociceptive aspects of pain. However, its application has to be performed under continuous cardiac monitoring.

The treatment options for NSF are based on case studies with limited numbers of patients. In the early days of NSF, the benefits of intense hemodialysis were discussed. In patients with end stage kidney disease approximately 98 % of the Gadolinium that freely circulate in the blood is removed with 3 hemodialysis cycles. Not more than 30 % of the Gadolinium chelates are removed with conventional dialysis techniques. Use of ultrapure dialysate and a high bicarbonate concentration combined with high flow for a duration of 5–6 h is said to achieve the best clearance rate [76]. There are some cases where a renal transplant and the resulting improved renal function lead to a decrease in the NSF symptoms. Among other discussed therapy options that were published are the extracorporal photopheresis, plasmapheris, UV-A1 Therapy, high dose intravenous Immunoglobulins, Imatinib Mesylate, Rabamycine and Pentoxifylline. The NSF registry additionally mentions some therapeutic regimens that were anecdotally reported, namely topical Dovonex, Cytoxan and Thalidomide. The use of oral steroids was discussed as well however the reports on the effectiveness were inconsistent (Table 2) [76-89].

**Table 2:** Overview of the examined treatment options

Established therapies	Physical Therapy
	Pain control (opoids, NSARs, steroids, antidepressants)
	Renal Transplantation
Based on case reports	Extracorporal photopheresis
	Plasmapheresis
	UV- A1 therapy
	High dose iv. Immunoglobulines
	Imatinib Mesylate
	Rabamycine
	Pentoxifylline
Anecdotal	Topical dovonex
	Cytoxan
	Thalidomide
	Immunosuppression via Steroids

Considering the small numbers of case reports and the fact that NSF can possibly be avoided by preventive measures, the focus of further attention should be primarily on prevention.

### Present guidelines in Europe and the USA - European Medicines Agency (2010), European Society of Urogenital Radiology (2008), FDA (2010) and American College of Radiology (2010)

The FDA was the first agency to release a public health advisory back in 2006 when the association of GBCA and NSF was not as convincing. However the linkage of high dose GBCA with NSF was suspected, accordingly the advisory stated that only if deemed necessary patients with advanced kidney failure (dialysis or eGFR < 15 ml/min/1.73 m²) should receive imaging studies using GBCA. An update in 2007 extended the patient collective at risk to patients with an eGFR of less than 30 ml/min/1.72 m² and also linked the induction of NSF to the hepato-renal syndrome and liver transplants. A screening of the renal function was proposed. GBCA dose should not exceed the FDA approved levels. In 2010 the latest FDA recommendations explicitly contraindicated Gadodiamide, Gadoversetamide and Gadopentetate dimeglumine use in patients with acute kidney disease or chronic severe kidney disease (eGFR<30). In general all GBCA should be avoided in patients with suspected or known impaired drug elimination unless the diagnostic GBCA based study is necessary. It is again stressed that the recommended dosage must not be exceeded and repeat dosing should be delayed until sufficient time has passed for the first dose to be completely eliminated. This might be less than one day for patients with normal renal function but up to a week for patients for severe chronic renal disease. The screening of the renal function and good documentation are also obligatory (Table 3) [90]. It should be also noted, that only little data on the safety of GBCAs for pediatric patients and almost no data for children younger than 2 years old is available.

**Table 3:** The FDA guidelines from 2006, 2007 and 2010 (www.fda.org)

**FDA 2006**	FDA 2007	FDA 2010
	Boxed Warning for GBCAs	Change in labeling for GBCAs
Patients at risk:	Patients at risk:	Patients at risk:
Moderate to end stage kidney disease (GFR < 60 ml/min/1.73 m²)	Acute or chronic severe renal insufficiency (GFR < 30 ml/min/1.73 m²) Acute renal insufficiency due to hepatorenal syndrome or in the perioperative phase of a liver transplant	Highest risk for patients with GFR < 30 ml/min/1.73 m²
		Repeated or high dosage GBCA enhanced procedures
		NO RISK: patients with normal kidney function
	Screen all patients for renal dysfunction (history and/or lab tests)	Screen all patients for renal dysfunction (history for assessment of acute kidney failure, lab tests for patients at risk for chronic renal failure) Risk factors are Diabetes, Hypertension, heart disease, smoking, obesity, high cholesterol, family history of kidney disease, age 65 or older, urinary tract infections/obstructions, systemic infections or autoimmune diseases [91]
Measures:	Measures:	Measures:
Carefully weigh pros and cons of GBCA enhanced imaging	avoid GBCA unless absolutely necessary	Gadodiamide, Gadoversetamid, Gadopentet Acid are contraindicated for highest risk patients
Use alternative imaging if possible	do not exceed recommended dose	
	take the elimination half life into account and allow enough time for GBCA elimination before rescanning the patient	
		Avoid GBCAs in patients with impaired (known/suspected) drug elimination unless absolutely necessary
Consider prompt dialysis in all patients with impaired renal function	Consider prompt dialysis in patients that are already on dialysis treatment	Consider prompt dialysis in patients that are already on dialysis treatment
		Monitor patients after GBCA administration, good documentation, no repeat GBCA imaging procedures until GBCA is eliminated from the body

The European Medicines Agency (EMA) has defined three risk classes of Gadolinium- based contrast agents. For each of them, the EMA has defined measures to reduce the risk of NSF.

1. High riskGadodiamide; Gadoversetamide, Gadopentetate Dimeglumine

2. Intermediate riskGadobenate Dimeglumine; Gadoxetate Disodium; Gadofosveset

3. Low riskGadobutrol, Gadoteridol; Gadoterate Meglumine

The EMEA’s recommendations on the use of GBCAs are based on this classification (see Table 4). However the concern about newborns is controversial because there are no known cases of NSF in any patient less than 6 years old in spite of a extensive use of high doses of gadolinium based contrast agents for evaluating congenital heart disease in this population [63]. The guidelines also point to the necessity of good documentation of contrast agent type and dose [92] which has not been reliable in the past (Table 4) [93].

**Table 4:** The EMA guidelines (www.ema.europa.eu)

Patients with severely impaired renal function	**high risk GBCAs** are contraindicated
	**medium and low risk GBCAs:** use lowest possible dose, pause at least 7d between two GBCA enhanced procedures
Patients with moderately impaired renal function	**High risk GBCAs**: use single injection of minimum dose, pause of 7d between two GBCA enhanced procedures
	**Medium and low risk GBCAs**: use minimum dose, pause of 7d between two GBCA enhanced procedures
Toddlers < 1yo	**High, medium and low risk GBCAs**: use single injection of minimum dose, pause of 7d between two GBCA enhanced procedures
Infants < 4wks	**high risk GBCAs** are contraindicated
	**medium and low risk GBCAs:** use lowest possible dose, pause at least 7d between two GBCA enhanced procedures
Breast feeding	**High risk GBCAs**: pause for 24 h
	**Medium and low risk GBCAs**: consider pause for 24 h
Perioperative phase of liver transplants	**high risk GBCAs** are contraindicated
	**medium and low risk GBCAs:** use lowest possible dose, pause at least 7d between two GBCA enhanced procedures

The European Society of Urogenital Radiology agrees with the EME on three risk classes for Gadolinium based contrast agents. Additionally, the ESUR defines three risk classes for patients. Patients with chronic kidney disease (CKD) stage 4 and 5 (eGFR < 30 ml/min), patients on dialysis and patients with impaired renal function and pending or received liver transplant are considered high risk patients. Patients with CKD stage 3 and small children younger than 1 year old have a lower risk and with regards to renal function healthy patients are at no risk for developing NSF. The EUSR guidelines also mention the treatment of pregnant women. Due to lack of experience, these patients are to be treated according to the protocol for infants [94].

Further preventative measures should be strived for. It is theorized that low iron serum levels, and an optimized Calcium, phosphate and acid base balance before injection of GBCAs may protect against the development of NSF [85]. Recent research has focused on the goal to limit GBCA doses as far as possible in order to still ensure good image quality. Some authors have reported sufficient image quality in 3 T scanners e.g. for abdominal imaging with 0,025 mmol/kg GBCA at 3 T [95], 0,05 mmol/kg for soft tissue characterization [96] and with 0,05 mmol/kg for vascular malformations [97]. Modifications in CE-MRA protocols offer further reduction of contrast agent doses [98].

### Guidelines of the American College for Radiology (2010)

The American College of Radiology (ACR) recommendations of 2010 agree with the other guidelines on the associations of GBCA exposure, dosage relations and kidney function. The need for adequately assessing the renal function has been highlighted. In the opinion of the ACR patients at risk, e.g. patients with a past medical history of a renal disease, aged above 60 years old, who have hypertension or diabetes mellitus, should be tested for their renal function 6 weeks prior the planned CMR study. Additionally, a verbal assessment might be helpful. The recommendations for the specific patient groups, divided by their renal function is listed in Table 5[99].

**Table 5:** Recommendations for specific patient groups by the American College of Radiology, 2010

Chronic Kidney Disease Stage 1 or 2	No special measures
(eGFR 60 to 119 ml/min/1.73 m^2^)	
Chronic Kidney Disease Stage 3a	Lowest possible dose of GBCA for diagnostic studies. If possible, avoid high risk GBCAs
(eGFR 45- 59 ml/min/1.73 m^2^)	
Chronic Kidney Disease Stage 3b	GBCA use should be safe if dosage is restricted to 0.1 mmol/kg or less. Apply same safety measures as in the following group
(eGFR 30- 44 ml/min/1.73 m^2^)	
Chronic Kidney Disease Stage 4 or 5 not on chronic dialysis	If feasible, avoid all contrast media. If usage is not avoidable, use lowest possible dose, avoid high risk GBCAs, avoid readministration of GBCAs for a week
(eGFR <30 ml/min/1.73 m^2^)	
End-stage renal disease with chronic dialysis	Consider change of imaging modality. Avoid high risk GBCAs, use lowest possible dose. **Schedule the imaging study as close as possible before the next routine hemodialysis session.**
(eGFR 45- 59 ml/min/1.73 m^2^)	
Acute kidney injury	If possible avoid any GBCA administration, regardless of eGFR. If GBCA use is necessary, avoid high risk GBCAs, use lowest possible dose.
Pregnancy	If possible, avoid use of GBCA, unless such an imaging study is absolutely necessary.
Pediatrics	Use similar safety measures as in adults. Neonates can have an eGFR of less than 30 ml/min/1.73 m³ but is not considered an absolute contraindication.

**Figure 2:**
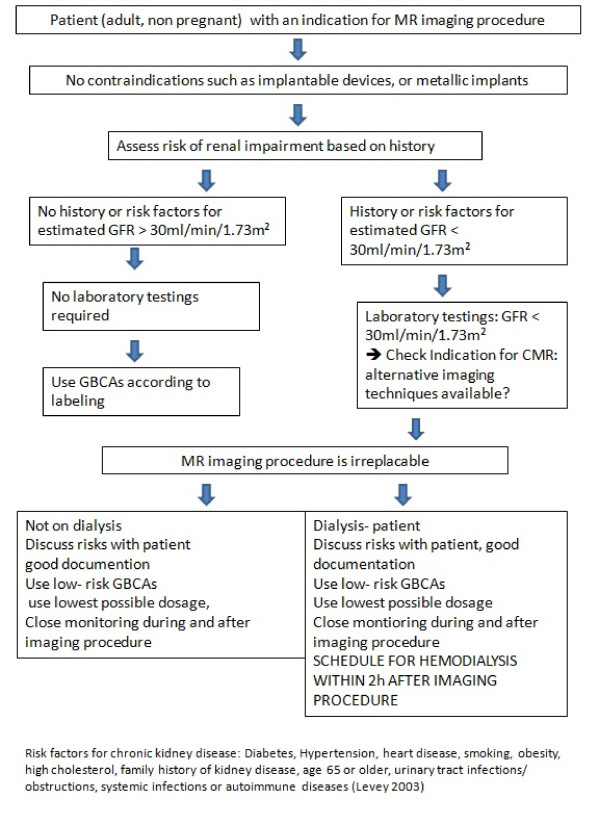
Work sheet “How to do it in adults”. **Work sheet “How to do it in adults”.** Among the risk factors for chronic kidney disease are Diabetes, Hypertension, heart disease, smoking, obesity, high cholesterol, racial factors, family history of kidney disease, ager 65 or older, urinary tract infections/obstructions, systemic infections or autoimmune diseases [91]. Standard doses for relevant procedures are 0.05-0.1 mmol/kg body weight for perfusion studies, 0.1-0.2 mmol/kg body weight for late gadolinium enhanced studies and 0.1-0.2 mmol/kg body weight for angiography studies [100,101].

The recommendations of the above mentioned guidelines can be incorporated into an easy to use short work sheet for non- pregnant adults for everyday use (Figure 2, Table 6: list of current label doses).

**Table 6:** **Current label doses for GBCAs**[102]

Gadoteridol	0.1 mmol/kg or 0.2 ml/kg
Gadoversetamide	0.1 mmol/kg or 0.2 ml/kg
Gadodiamide	CNS: 0.1 mmol/kg or 0.2 ml/kg
	Body: 0.05 mmol/kg or 0.1 ml/kg
	Intrathoracic/intraabdominal/pelvic: 0.1 mmol/kg or 0.2 ml/kg
Gadobenate dimeglumine	0.1 mmol/kg or 0.2 ml/kg
Gadopentetate dimeglumine	0.1 mmol/kg or 0.2 ml/kg
Gadofosveset	0.12 mmol/kg or 0.03 ml/kg
Gadoxetate disodium	0.025 mmol/kg or 0.1 ml/kg
Gadoterate meglumine	0.1 mmol/kg or 0.2 ml/kg
Gadobutrol	0.1 mmol/kg or 0.1 ml/kg

## Conclusions

NSF is one of the major risks associated with GBCA enhanced CMR procedures. Many of its features and pathogenetic characteristics have been already revealed, however important aspects still have to be focused on, for example the treatment of NSF. For the time being, the treatment is limited to symptomatic relieve and prevention. Several up to date guidelines are available, explaining the aspects of a safe GBCA enhanced CMR procedure. The benefits of GBCA enhanced CMR techniques are many in numbers, and should not be withheld rashly. Even with the lack of new NSF cases, the general awareness for NSF has to be strengthened in order to prevent the reoccurrence of this GBCA side effect.

## Misc

This work has been funded in parts by the Bavarian research foundation

## Competing interests

MRP: patent agreements with Bayer, Bracco, GE Healthcare, Mallinckrodt, Lantheus and Epix.

EN: Significant research support: Bayer Healthcare, all others have no conflicts of interest.

## Authors’ contributions

TR carried out the review of literature, manuscript design and drafting. OR, MRP, PN, CW has been involved in revising the manuscript critically for important intellectual content. EN and WRB has been involved in drafting the manuscript and participated in manuscript design and revision. All authors read and approved the final manuscript.
